# Systemic immune dysregulation in patients experiencing urogynaecological mesh failure

**DOI:** 10.1002/bco2.70210

**Published:** 2026-04-30

**Authors:** Shannon Jamieson, Cameron R. Dougan, Eloise Rennie, Patrick Card, Sudarshan Nallappa, Karen Smith, David J. Deehan, Karen Brown, Catharien M. U. Hilkens, Christopher Harding

**Affiliations:** ^1^ Translational and Clinical Research Institute Newcastle University Newcastle upon Tyne UK; ^2^ Newcastle Hospitals NHS Foundation Trust Newcastle upon Tyne UK

**Keywords:** cell migration, chemotaxis, immune system, inflammation, lower urinary tract symptoms, mesh

## Abstract

**Objectives:**

This study aimed to investigate the systemic immune landscape in patients experiencing urogynaecological mesh failure. The use of urogynaecological mesh was paused in the UK following an independent safety review that found ~1 in 15 women required removal due to complications. However, the mechanisms underpinning mesh failure remain largely unknown, with few studies focussing on localised tissue responses and no reports characterising systemic immune dysregulation.

**Materials and methods:**

Serum samples collected from patients during mesh removal surgery were analysed for immunomodulatory protein content using enzyme‐linked immunosorbent assay (ELISA) and multiplex Luminex Discovery Assay. Peripheral blood mononuclear cells (PBMCs) collected were cultured with or without exposure to pristine mesh, and immunomodulatory protein secretion was measured in the same way. Patient serum was also used as a migratory stimulus for healthy PBMCs to test the functionality of chemotactic proteins present.

**Results:**

Mesh patient serum had increased chemotactic protein levels, particularly CCL2, CXCL5, CCL12 and CCL4, which had a functional effect and induced significant cell migration. Mesh patient PBMCs also secreted immunomodulatory proteins including MMP‐9 and CCL2. Future studies should focus on expanding cohort numbers and including a control group of mesh patients not experiencing complications to further determine both underlying biology and mesh responses.

**Conclusions:**

This study characterised an altered systemic immune landscape not previously investigated in mesh failure patients. Clinically, the ability to identify phenotypic factors or develop biomarkers which predict and monitor mesh responses would help guide clinicians and patients in shared decision making.

## 1 INTRODUCTION

Stress urinary incontinence (SUI) and pelvic organ prolapse (POP) are common pelvic floor conditions which affect approximately 30%–40% of women worldwide.^1^, ^2^ Both conditions can result from weakened pelvic floor muscles and can greatly impact patient quality of life. Surgical intervention is usually indicated where conservative treatment such as pelvic floor physiotherapy and lifestyle changes has failed and includes a range of procedures using polypropylene mesh. Urogynaecological meshes were first used for the treatment of SUI and POP several decades ago and have also been widely used for hernia repair.^3^ Meshes for SUI are placed under the mid‐urethra in order to restore its normal retropubic position and alleviate symptoms including urinary incontinence.^4^ In POP, on the other hand, the mesh can be anchored to pelvic structures in order to support pelvic organs and relieve symptoms such as pain, pressure or bulging into the vagina.^5^ Initial successes with polypropylene mesh for the treatment of SUI and POP led to the first FDA‐approved meshes coming to market in the late 1990s. The NHS reports that between 2008 and 2017, approximately 127 000 women underwent a mesh insertion procedure for SUI or POP.^6^

Despite estimated success rates close to 90%,^7^, ^8^ concerns were raised over the safety of urogynaecological mesh implants by patient advocacy groups which led to extensive media attention. As a result of patient and professional testimonies from a series of NHS inquiries, an independent safety review was ordered by the UK Government in 2018. The review reported that 1 in 15 women may come to need surgical removal of urogynaecological mesh due to complications and raised concerns over the impact on patient quality of life.^9^ Complications reported by patients include chronic pain, recurrence of symptoms, mobility issues, infection, and mesh exposure.^10^ Additionally, the review highlighted the impact on mental health associated with lack of care provision for these mesh complications and cited significant levels of depression and anxiety. An immediate pause was placed on mesh insertion surgery in the UK in 2018 and the establishment of specialist centres for the evaluation and treatment of mesh‐related complications followed. Although mesh insertion for urogynaecological conditions is paused in the UK, the effects of previously inserted meshes continue to impact women.

While the literature pertaining to urogynaecological mesh complications remains limited, some studies have focussed on the role of the immune system. Although mild inflammatory responses are necessary for integration of biomaterials into host tissue,^11^ unresolved inflammatory responses can lead to chronic inflammation and this may contribute to the development of mesh complications. A cohort study of 27 explanted meshes demonstrated an increased macrophage infiltrate in patient tissue surrounding mesh fibres on immunofluorescence microscopy and a shift in pro‐inflammatory chemokine secretion towards TNF‐α and CXCL10.^12^ Similarly, a study examining 100 polypropylene meshes explanted from the pelvic floor found increased macrophage, lymphocyte, and neutrophil infiltrate in explanted mesh tissue which correlated with material degradation.^13^ The role of lymphocytes has also been investigated in a cohort study of 42 patients experiencing mesh exposure and showed significantly increased CD4+, CD8+ and FoxP3+T cells in explanted mesh tissue.^14^ Together these findings indicate a role of both localised innate and adaptive immune cells in mesh complications in terms of the peri‐implant space; however, no reported studies have investigated the role of the systemic immune response.

Although patients experiencing mesh complications tend to report localised symptoms such as abdominal pain and urinary issues, systemic symptoms such as dry eyes, dental issues, joint pains and hair loss have also been reported anecdotally.^9^, ^15^ Degradation of polypropylene mesh is hypothesised to contribute to the development of systemic immune responses and may lead to complications in patients with a predisposition for autoimmune diseases.^16^ Additionally, studies using animal models have demonstrated the ability of polypropylene particles to remain in tissue even following the removal of mesh implants.^17^ Together these findings suggest a role for the immune system in mesh complications which do not remain localised and have the potential to mediate systemic effects. However, no studies have investigated systemic immune responses to polypropylene mesh or compared mesh failure patients to those undergoing procedures for nonmesh indications. Due to the paucity in the literature, this study therefore investigated the systemic immune landscape in urogynaecological mesh failure.

## 2 METHODS

### 2.1 Patient sample collection and processing

Patients undergoing surgery for urogynaecological mesh removal or recovery (*n* = 12) or POP (*n* = 4) were invited to take part in the Tissue and Explant Analysis of Medical Implant Failure (TEAM) study following NHS REC approval (20/LO/2012). All participants provided written informed consent in accordance with the NHS Research Authority. Sex‐matched healthy control peripheral whole blood was collected as part of the LimeTrees study at Newcastle University under AWERB Project ID No 633 (*n* = 6). Peripheral whole blood samples were collected in Vacuette K3EDTA (BD Biosciences, USA) and Vacuette serum clot activator (BD Biosciences) tubes.

Serum separation tubes were incubated at room temperature for 30 min before being centrifuged at 1800*g* for 12 min at room temperature. Once separated, serum was stored at −80°C.

Peripheral whole blood was diluted 1:1 with room temperature phosphate‐buffered saline (PBS) supplemented with 2‐mM EDTA and layered over Lymphoprep™ (Stemcell technologies) at a ratio of 2:1. Density centrifugation was performed for 30 min at 900*g* at room temperature. Peripheral blood mononuclear cells (PBMCs) were collected from the serum‐Lymphoprep™ interface and added to cold PBS supplemented with 1% foetal calf serum (FCS). Samples were centrifuged at 600*g* at 4°C for 8 min. Pellets were resuspended before centrifugation at 300*g* at 4°C for 8 min to remove platelets. Pellets were resuspended and strained through a 70‐μm nylon filter (Greiner, Germany) then counted. PBMCs were then centrifuged at 400*g* at 4°C for 8 min and resuspended in FCS supplemented with 10% dimethylsulfoxide (DMSO) and stored at −80°C for 24 h before transfer to liquid nitrogen for long‐term storage.

### 2.2 Mesh re‐exposure

Stored autologous serum was thawed and added at a concentration of 2% to RPMI‐1640 medium supplemented with 50 U/mL of penicillin, 50 μg/mL og streptomycin and 2 mM of L‐glutamine (all Sigma‐Aldrich, UK). Stored PBMCs were thawed and centrifuged at 300*g* for 5 min; pellets were resuspended in blank RPMI‐160 medium for 1 h. 1 × 10^6^ PBMCs were exposed to a 1‐cm^2^ square of sterile unused polypropylene mesh (Praxisdienst, Germany) for 24 h. Untreated cells were used as a negative control, and lipopolysaccharide (LPS from *Escherichia coli* 0111:B4) (Sigma‐Aldrich) was used at a concentration of 10 ng/ml as a positive control. Following a 24‐h exposure, cell supernatant was collected and stored at −20°C for use in subsequent assays.

### 2.3 Protein secretion analysis

Cell supernatants and serum were used in conjunction with Human DuoSet enzyme‐linked immunosorbent assay (ELISA) kits (R&D Systems, USA) in accordance with the manufacturer's instructions. Cell supernatants and serum (2% in RPMI‐1640) were then used in conjunction with a multi‐plex Luminex Discovery Assay (R&D Systems) to measure the levels of additional proteins not examined using ELISA according to the manufacturer's guidance.

### 2.4 PBMC migration

Active cellular migration was assessed using a Millicell insert (Merck, Germany) with a pore size of 3‐μm inside a 24‐well tissue culture plate. Healthy PBMCs were isolated from a cone (NHSBT) and added at a density of 4 × 10^5^ to the upper chamber. About 2% serum in RPMI‐1640 was added to the lower chamber. A chemokine cocktail consisting of CXCL10 and CXCL12 was used as a positive migratory stimulus, and blank RPMI‐1640 was used as a negative control. PBMC migration was assessed following a 2‐h incubation at 37°C by harvesting the cells in the lower chamber, counting them and calculating a migration efficiency as a percentage of PBMCs input. Millicell inserts were submerged in cold methanol and stored at −20°C overnight. The inserts were then stained with haematoxylin (Sigma‐Aldrich) for 5 min, blued in 0.2% ammonia water and dehydrated through graded alcohols (70%–99%). Each insert was air‐dried for 3 h before the filter was cut out and mounted in Pertex mounting medium (Histolab, Sweden) and imaged.

### 2.5 Statistical analysis

Statistical analysis was carried out using GraphPad Prism 10.4.2 using analysis of variance (ANOVA) with Dunnett's multiple comparisons unless otherwise stated. Significance level was set at *p* < 0.05. All error bars represent standard error of the mean (SEM).

## 3 RESULTS

### 3.1 Patient demographics

The demographics including primary reason for surgery are shown in Table 1. The average age for healthy controls was notably lower than the other groups at 27 years compared to 74 and 59 years in the nonmesh and mesh groups respectively. The average time from mesh implantation to surgery was 13 years, and 8 of the 12 mesh patients cited pain as an ongoing complication (66.6%). Mesh erosion was noted in 3 of the 12 patients (25%). Ten of the 12 patients underwent surgery for a full mesh removal, 1 patient had a partial mesh removal and 1 patient had a mesh recovering where the implant was not removed. One patient, M50, also reported Graves' disease; however, no other autoimmune conditions were noted within the patient cohort. In the nonmesh cohort, 100% of patients underwent surgery for repair of POP with an equal split between cystocele (2/4 patients) and rectocele (2/4 patients). This was achieved using native tissue to allow for sacrospinous or uterosacral ligament suspension.

**TABLE 1:** Table of patient demographics.

Group	Patient ID	Age (years)	Reason for mesh insertion	Surgery	Reason for removal	Implantation time (years)
Healthy control	HC1	28	N/A	N/A	N/A	N/A
	HC2	25	N/A	N/A	N/A	N/A
	HC3	24	N/A	N/A	N/A	N/A
	HC4	28	N/A	N/A	N/A	N/A
	HC5	27	N/A	N/A	N/A	N/A
	HC6	27	N/A	N/A	N/A	N/A
Nonmesh	M43	78	N/A	Pelvic floor reconstruction	N/A	N/A
	M45	79	N/A	Pelvic floor reconstruction	N/A	N/A
	M46	82	N/A	Pelvic floor reconstruction	N/A	N/A
	M57	55	N/A	Pelvic floor reconstruction	N/A	N/A
Mesh	M37	57	POP	Mesh removal	Pain, SUI	14
	M38	52	SUI	Mesh removal	Pain, SUI, thrush	16
	M39	58	POP	Mesh removal	Recurrent UTI, pain, inflammation	15
	M40	61	POP	Mesh removal	Pain	26
	M47	39	Grade II endometriosis	Mesh removal	Pain	10
	M49	60	SUI	Partial mesh removal	SUI	9
	M50	61	SUI	Mesh removal	SUI	10
	M51	57	Unknown	Mesh recovery	Mesh erosion, pain	14
	M52	77	POP	Mesh removal	Pain	13
	M53	58	POP	Mesh removal	Mesh erosion	9
	M54	57	SUI	Mesh removal	Mesh erosion, pain	Unknown
	M56	68	SUI	Mesh removal	SUI	11

### 3.2 Altered immune secretion profile of mesh patient PBMCs

**FIGURE 1:**
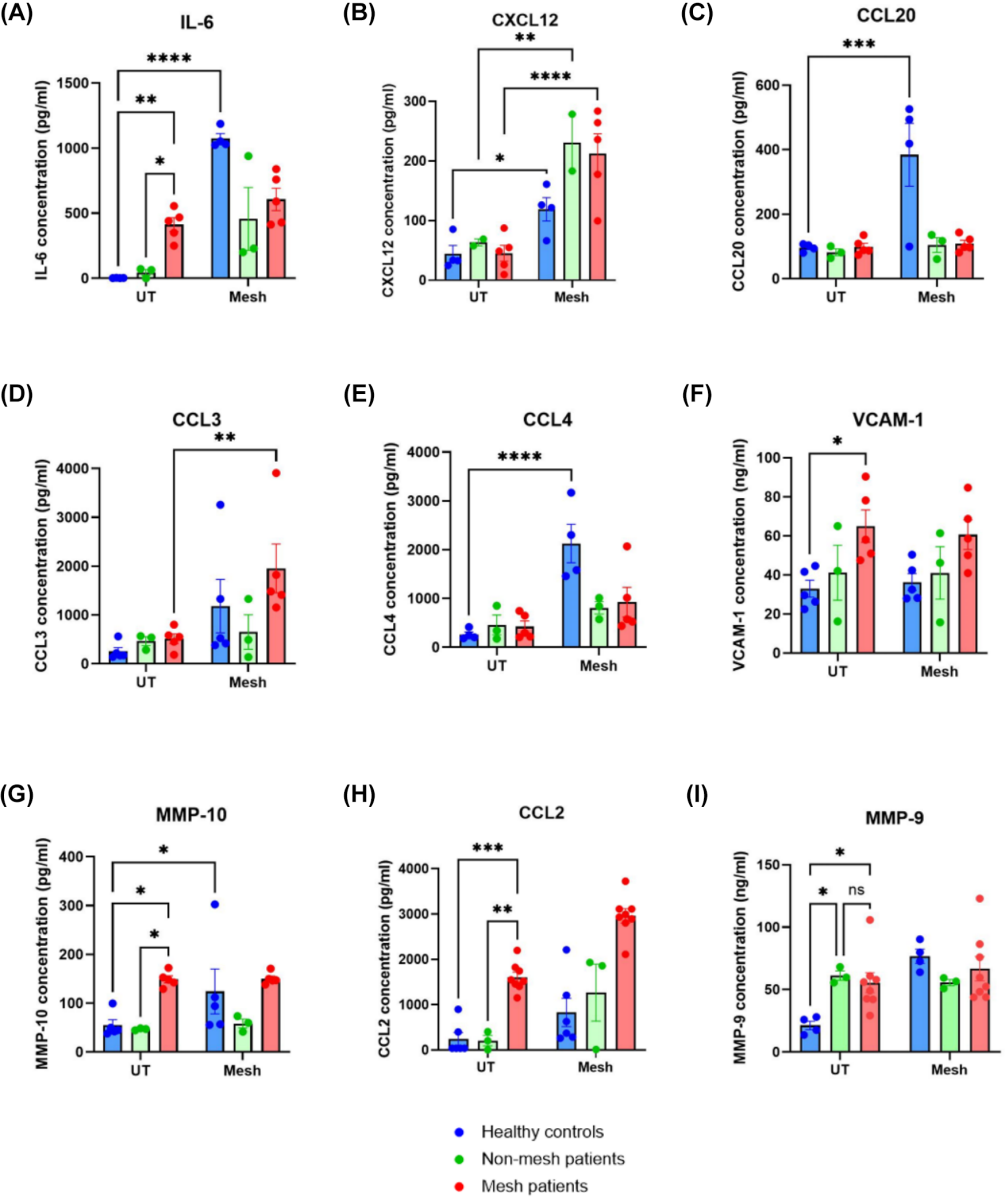
Secretion of immunomodulatory proteins from PBMCs exposed to mesh. PBMCs were cultured for 24 h with or without exposure to polypropylene mesh, and 10 ng/mL LPS was used as a positive control. Cell supernatants were used to measure the secretion of immunomodulatory proteins (A–G) using a Luminex multiplex discovery assay. Supernatants were also used in conjunction with human DuoSet ELISA kits to quantify CCL2 (H) and MMP‐9 (I) levels and validate the ex vivo cell stimulation method. (A) IL‐6 secretion from mesh patient PBMCs was significantly increased when compared to both healthy controls and nonmesh patients (*p* = 0.0080 and *p* = 0.0291). Upon re‐exposure to mesh, healthy control PBMCs significantly increased their secretion of IL‐6 (*p* < 0.0001). (B) Although no significant differences in CXCL12 were observed in the untreated PBMCs, upon re‐exposure to mesh all groups experienced significant increases in secretion (healthy controls *p* = 0.0442, nonmesh *p* = 0.0032 and mesh patients *p* < 0.0001). (C) CCL20 and (E) CCL4 secretion was significantly increased in healthy control PBMCs upon mesh re‐exposure (*p* = 0.0001 and *p* < 0.0001); however, no other notable changes were observed. (D) CCL3 secretion was significant increased from mesh patient PBMCs once re‐exposed to mesh (*p* = 0.0083) but not when compared to healthy controls. (F) VCAM‐1 and (G) MMP‐10 secretion was significantly increased by mesh patient PBMCs compared to healthy controls (*p* = 0.0168 and *p* = 0.0157). However, MMP‐10 was also significantly increased by mesh patient PBMCs when compared to the nonmesh group (*p* = 0.0233). Additionally, a significant increase in MMP‐10 secretion was observed in the healthy control group upon exposure to mesh (*p* = 0.0348). (H) A significant increase in CCL2 secretion from untreated PBMCs was observed in the mesh group when compared to both healthy controls (*p* =*p* = 0.0004) and nonmesh patients (*p* = 0.0039). Mesh patient PBMCs which were re‐exposed to mesh also experienced a significant increase in CCL2 secretion compared to untreated (*p* = 0.0002). (I) MMP‐9 secretion was significantly increased by untreated mesh patient PBMCs when compared to healthy controls (*p* = 0.0149) but not nonmesh patients (*p* = 0.8970). Untreated nonmesh PBMCs secreted significantly more MMP‐9 than healthy controls (*p* = 0.0239). Only healthy control PBMCs were capable of being stimulated to produce MMP‐9 through mesh exposure (*p* = 0.0006). Statistical analysis was performed using a two‐way ANOVA with Dunnett's multiple comparisons; error bars represent the standard error of the mean (SEM).

To assess systemic immune activation of mesh patients and validate ex vivo exposure of PBMCs to polypropylene mesh, ELISA was used to determine inflammatory protein levels (Figure 1H,I). Secretion of CCL2 and MMP‐9 from untreated PBMCs was significantly increased in mesh patients when compared to healthy controls (*p* = 0.0004 and *p* = 0.0149). CCL2 secretion was also significantly increased when compared to the nonmesh patient cohort (*p* = 0.0039). MMP‐9 secretion, on the other hand, was not significantly different in the mesh and nonmesh patient cohorts, but there was a significant increase observed when comparing nonmesh patients to healthy controls (*p* = 0.0239). Interestingly, upon re‐exposure to mesh, there was a significant increase in CCL2 secretion from mesh patient PBMCs (*p* = 0.0002), whereas MMP‐9 secretion significantly increased from healthy control PBMCs following exposure to mesh (*p* = 0.0006).

### 3.3 Increased levels of chemotactic factors in mesh patient serum

Following ELISA, 12 immunomodulatory proteins were assessed using a Luminex Discovery Assay. Six cytokines were selected based on the observed changes to CCL2 (Figure 1A–E). Of the six cytokines investigated, all with the exception of CXCL5 showed significant differences in secretory levels. IL‐6, CXCL12 (*p* = 0.0442), CCL3 and CCL4 were significantly upregulated by healthy control PBMCs following mesh exposure. CXCL12 secretion was also significantly increased in the nonmesh (*p* = 0.0032) and mesh groups (*p* < 0.0001) when compared to untreated. Only IL‐6 secretion from mesh patient PBMCs was significantly increased when compared to both healthy controls (*p* = 0.0080) and nonmesh patients (*p* = 0.0291) in the untreated group.

Six other immunomodulatory proteins were assessed, and the only notable differences observed were in the secretion of VCAM‐1 and MMP‐10 (Figure 1H,I). Secretion of VCAM‐1 (*p* = 0.0168) and MMP‐10 (*p* = 0.0157) was significantly increased in the untreated mesh patient group compared to healthy controls. Interestingly, MMP‐10 secretion was also significantly upregulated when comparing untreated mesh and nonmesh groups (*p* = 0.0233). Exposure of healthy control PBMCs to mesh also resulted in a significant increase in MMP‐10 secretion (*p* = 0.0348). No notable differences were observed in the remaining immunomodulatory proteins investigated both at untreated and upon exposure to mesh (Supporting Information S1).

**FIGURE 2:**
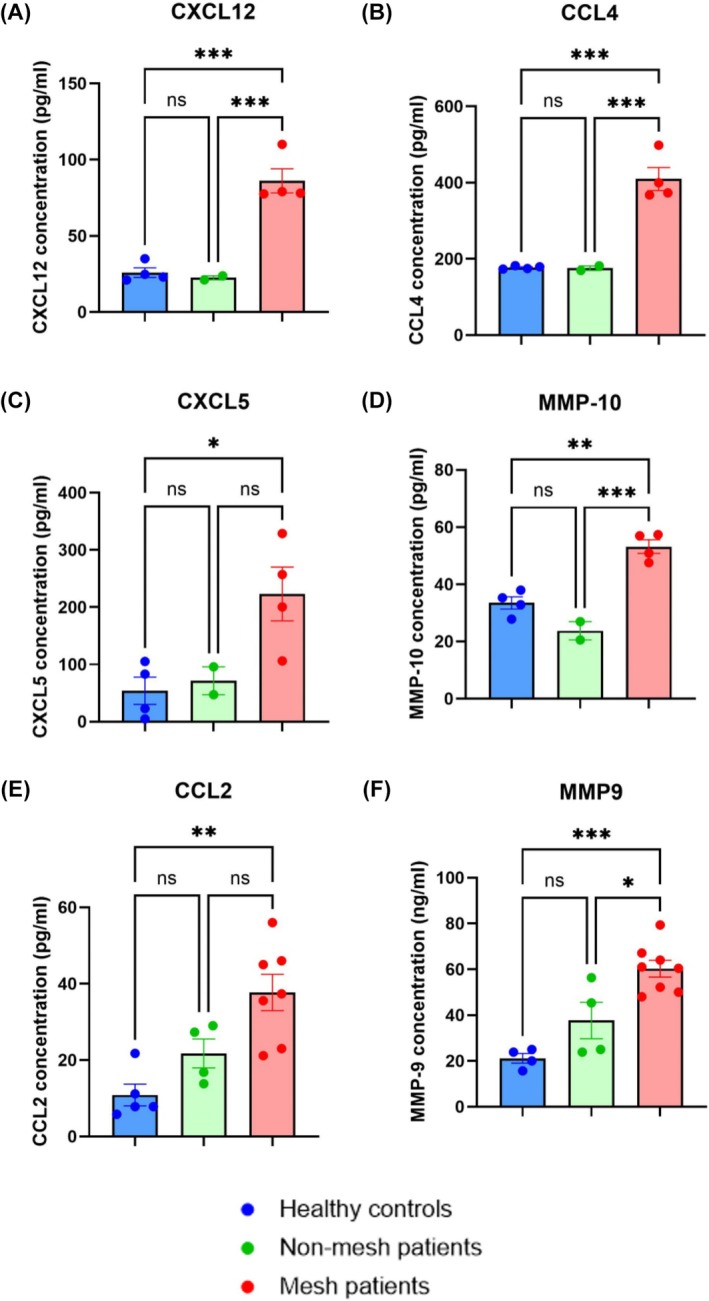
Serum levels of immunomodulatory proteins. RPMI‐1640 was supplemented with 2% serum from healthy controls, mesh patients or nonmesh patients and used in conjunction with a Luminex Discovery Assay to measure the levels of immunomodulatory proteins (A–D) Serum was also used with a human DuoSet ELISA kits to quantify CCL2 (E) and MMP‐9 (F) levels. (A) Levels of CXCL12 in mesh patient serum was significantly increased when compared to both nonmesh serum (*p* = 0.0008) and healthy control serum (*p* = 0.0003). There was no significant difference in CXCL12 levels between healthy controls and nonmesh patients observed (*p* = 0.9339). (B) CCL4 levels were significantly higher in mesh patient serum when compared to both healthy controls (*p* = 0.0002) and nonmesh patients (*p* = 0.0007). No significant difference was observed between healthy controls and nonmesh patients (*p* = 0.9988). (C) CXCL5 levels were significantly increased in mesh patient serum compared to healthy controls (*p* = 0.0271). There was no significant differences between observed between CXCL5 levels in mesh patient serum when compared to nonmesh patients (*p* = 0.0934) or between nonmesh and healthy control serum (*p* = 0.9559). (E) CCL2 levels were significantly increased in mesh patient serum compared to health controls (*p* = 0.0013) but not when compared to nonmesh patients (*p* = 0.0570). Similarly, no statistical difference was seen between healthy controls and nonmesh patient serum levels of CCL2 (*p* = 0.2684). (F) Levels of MMP‐9 in mesh patient serum were significantly higher than both nonmesh patients (*p* = 0.0131) and healthy controls (*p* = 0.0002). Statistical analysis was performed using a one‐way ANOVA with Dunnett's multiple comparisons; error bars represent the standard error of the mean (SEM).

The same 12 immune‐related proteins used for protein secretion analysis were also used to assess levels of protein in serum (Figure 2A–D). Interestingly, the only significant differences observed were in chemotactic proteins CXCL5 (*p* = 0.0271), CXCL12 (*p* = 0.0003) and CCL4 (*p* =*p* = 0.0002) when comparing mesh patient serum to healthy controls. CXCL12 and CCL4 levels were also significantly higher in the mesh patient group when compared to nonmesh (*p* = 0.0008 and *p* = 0.0007, respectively). Similarly, ELISA showed serum levels of CCL2, and MMP‐9 levels were significantly increased in the mesh patients compared to healthy controls (*p* = 0.0204 and *p* = 0.0002) (Figure 2E,F). MMP‐9 levels were significantly higher in mesh patient serum when compared to nonmesh (*p* = 0.0131). No other notable differences were observed in serum levels of the proteins investigated (Supporting Information S2).

### 3.4 Mesh patient serum can act as a migratory stimulus

**FIGURE 3:**
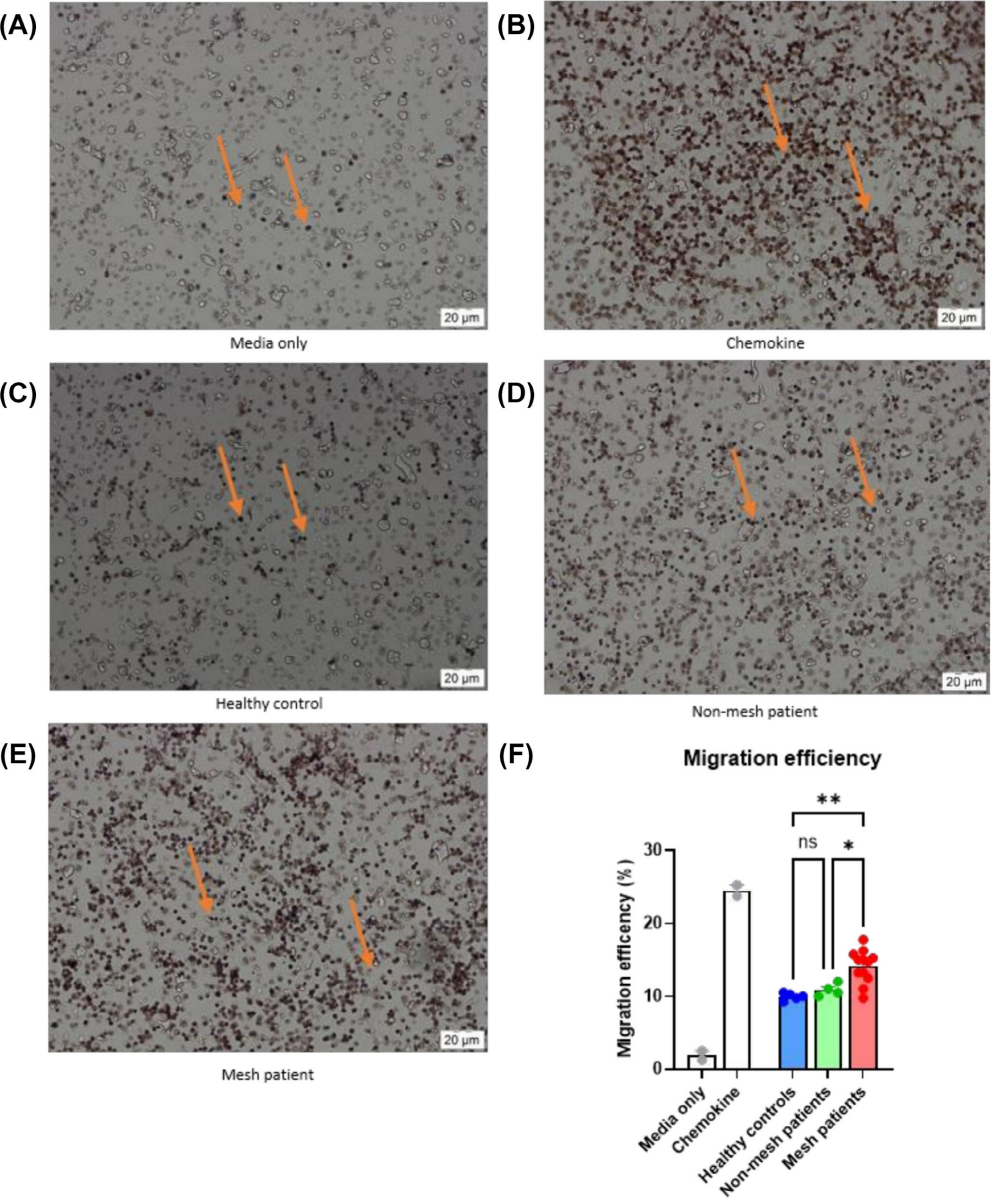
Migratory effect of serum on PBMCs. Healthy PBMCs were added to the upper chamber of a Millicell insert with a 3‐μm pore size; RPMI‐1640 supplemented with 2% serum was added to the bottom chamber. Unsupplemented RPMI‐1640 media (media only) was used as a negative control, and a chemokine cocktail containing CXCL10 and CXCL12 was used as a positive migratory stimulus. Millicell inserts were stained with haemotoxylin to visualise migrated cells and migration efficiency was calculated by harvesting cells which were present in the lower chamber. (A) Media only without serum added resulted in few PBMCs migrating through the Millicell insert. (B) A larger proportion of PBMCs migrated through the Millicell insert towards the chemokine cocktail. (C) Serum from healthy control patients resulted in the migration of a small proportion of PBMCs relative to mesh patients. (D) Comparable migration of PBMCs was noted towards nonmesh patient serum when compared to healthy control. (E) Mesh patient serum resulted in a notable increase in PBMC migration when compared to healthy controls. (F) Migration efficiency of healthy PBMCs with mesh patient serum as a stimulus was significantly increased when compared to both nonmesh patient serum (*p* = 0.0165) and healthy control serum (*p* = 0.0012). There was no statistical difference in the migratory efficiency of PBMCs comparing healthy control serum and nonmesh patient serum as a stimulus (*p* = 0.7332). Statistical analysis was performed using a one‐way ANOVA with Dunnett's multiple comparisons; error bars represent the standard error of the mean (SEM).

Due to the increased levels of chemotactic proteins present in mesh patient serum, a migration assay was used to determine whether a functional effect could be observed (Figure 3). A notably higher number of migrated cells could be observed towards the mesh patient serum than both the nonmesh and healthy control serum (Figure 3C–E). A significant increase in migration efficiency of cells was observed in response to mesh patient serum compared to both nonmesh patients and healthy controls, and no significant difference in migration efficiency was observed between healthy control and mesh patient serum (Figure 3F).

## 4 DISCUSSION

Due to the very limited literature but growing anecdotal evidence amongst patients,^15^ this study investigated the systemic immune landscape in urogynaecological mesh complications. We demonstrate systemic immune dysregulation characterised by significant increases in immunomodulatory protein production from untreated mesh patient PBMCs as well as increased levels of chemotactic factors present in mesh patient serum. Both healthy controls and nonmesh patients were used as comparators throughout the study in order to gain a greater understanding of the effect of urogynaecological conditions and mesh implants while also taking into account age.

Importantly, there is growing concern pertaining to the systemic effects of urogynaecological implants highlighted through patient reports and increased media attention.^9^ Our study presents the first investigation of systemic immunomodulatory protein levels in patients experiencing urogynaecological mesh complications and shows significant changes in the serum levels of these proteins as well as the levels that are secreted by the patient's own immune cells. Similar to the findings in other implant types, IL‐6, MMP‐9 and CCL2 have been implicated in host immune responses to replacement joints and have been shown to be increased in patient synovial fluid and tissue taken from failed orthopaedic implants.^18^, ^19^, ^20^ Interestingly, mesh patient PBMCs were capable of being activated by re‐exposure to mesh and this resulted in significant secretion of chemotactic proteins CCL2, CCL3 and CXCL12 which are known to induce immune cell migration during medical implant failure.^21^ Healthy control PBMCs were more activated by mesh exposure, and this led to significant increases in the secretion of MMP‐9, MMP‐10, CXCL12, CCL20 and CCL4. This disparity between groups may be due to the readiness of PBMCs to be activated, as cellular exhaustion may have been reached by those already readily secreting higher concentrations of protein. Additionally, other factors such as age may play a role in these responses as the immune system adapts over time.^22^ Interestingly, nonmesh patient cells did not appear to be activated by mesh, and this may be due to a combination of cellular exhaustion as well as the lack of exposure to polypropylene experienced by the patients. Moreover, significantly higher levels of protein were observed in mesh patient serum which included CCL2, MMP‐9, CXCL5, CXCL12 and CCL4 when compared to healthy controls. Additionally, MMP‐9, CXCL12 and CCL4 were also significantly increased when comparing mesh patients to nonmesh. These data show that patients experiencing urogynaecological mesh failure are in a more pronounced inflammatory state systemically than their nonmesh counterparts. Interestingly, patient M50 had a reported diagnosis of Grave's disease; however, this autoimmune condition did not appear to impact their PBMC responses or serum chemokine levels when compared with other mesh patients. Future studies with expanded participant numbers could perform subgroup analysis according to autoimmune status in order to further understand whether a predisposition to autoimmunity impacts urogynaecological mesh outcomes.

Due to the pronounced chemokine levels measured, mesh patient serum was also tested as a migratory stimulus. No notable differences were observed in the migration of PBMCs towards healthy control and nonmesh patient serum, which reflects the findings from the protein quantification assays performed. Migration efficiency of PBMCs towards mesh patient serum, on the other hand, was significantly increased when compared to both nonmesh patients and healthy controls. This demonstrates the functional ability of the chemokines quantified in this study and provides a vital link between the reported systemic side effects by patients with the cellular infiltrate published in the literature.^12^, ^13^, ^14^

Overall, this study demonstrates an altered systemic immune landscape which has not previously been characterised in patients experiencing urogynaecological mesh failure; at this stage, it remains unclear whether the mesh failure causes this or is a result of pre‐existing conditions. Further investigations should aim to address the caveats of this study, in particular, expanding analyses into a cohort of patients who have an implanted urogynaecological mesh but are not experiencing any adverse reactions and therefore do not require removal. Additionally, increased patient numbers would allow for subgroup analysis comparing true mesh failure such as erosion and exposure compared to natural progression of pelvic floor dysfunction. Clearly future studies expanding patient numbers are needed to confirm the relationships we have reported. Additionally, the inclusion of age‐matched healthy controls would allow for further understanding of the role of age on the responses investigated in this study. Finally, analysis of the explanted mesh has not been carried out in this study, but investigations have shown polypropylene degradation resulting in polymer particle release^23^ which could be incorporated into future mesh failure studies in order to explore possible relationships between immune system activation and mechanical failure of mesh.

Clinically, it would be useful to identify phenotypic factors that may predict mesh complications and studies such as ours are a step towards this goal. There is no doubt that mesh surgery is an effective treatment for SUI and POP,^2^, ^24^ but it is also clear that some women suffer significant complications from these procedures.^25^ The ability to identify those patients more likely to experience adverse outcomes would advance the surgical management of SUI and POP by aiding the pre‐operative counselling and shared decision‐making processes.

## AUTHOR CONTRIBUTIONS

Shannon Jamieson: conception of study, data acquisition and analysis, manuscript drafting and editing. Cameron R Dougan: data acquisition and analysis. Eloise Rennie: data acquisition and analysis. Patrick Card: patient sample acquisition. Sudarshan Nallappa: patient sample acquisition. Karen Smith: administrative support. David J Deehan: acquisition of funding and study conceptualisation. Karen Brown: patient sample acquisition and clinical study support. Catharien MU Hilkens: study conceptualisation, critical review of manuscript. Christopher Harding: patient sample acquisition, conceptualisation of study, clinical data acquisition, critical review of manuscript.

## CONFLICT OF INTEREST STATEMENT

No conflicts exist.

## Supporting information


**Data S1.** Supporting information.
