# Supplementary material for: Systemic immune dysregulation in patients experiencing urogynaecological mesh failure
Source: BJUI Compass. 2026 Apr 30;7(5):e70210. doi: 10.1002/bco2.70210 (PMC13133428; doi:10.1002/bco2.70210)
Supplement: Supplementary file 1 — Data S1. Supporting information. [file BCO2-7-e70210-s001.docx]

**Supplementary material**

**
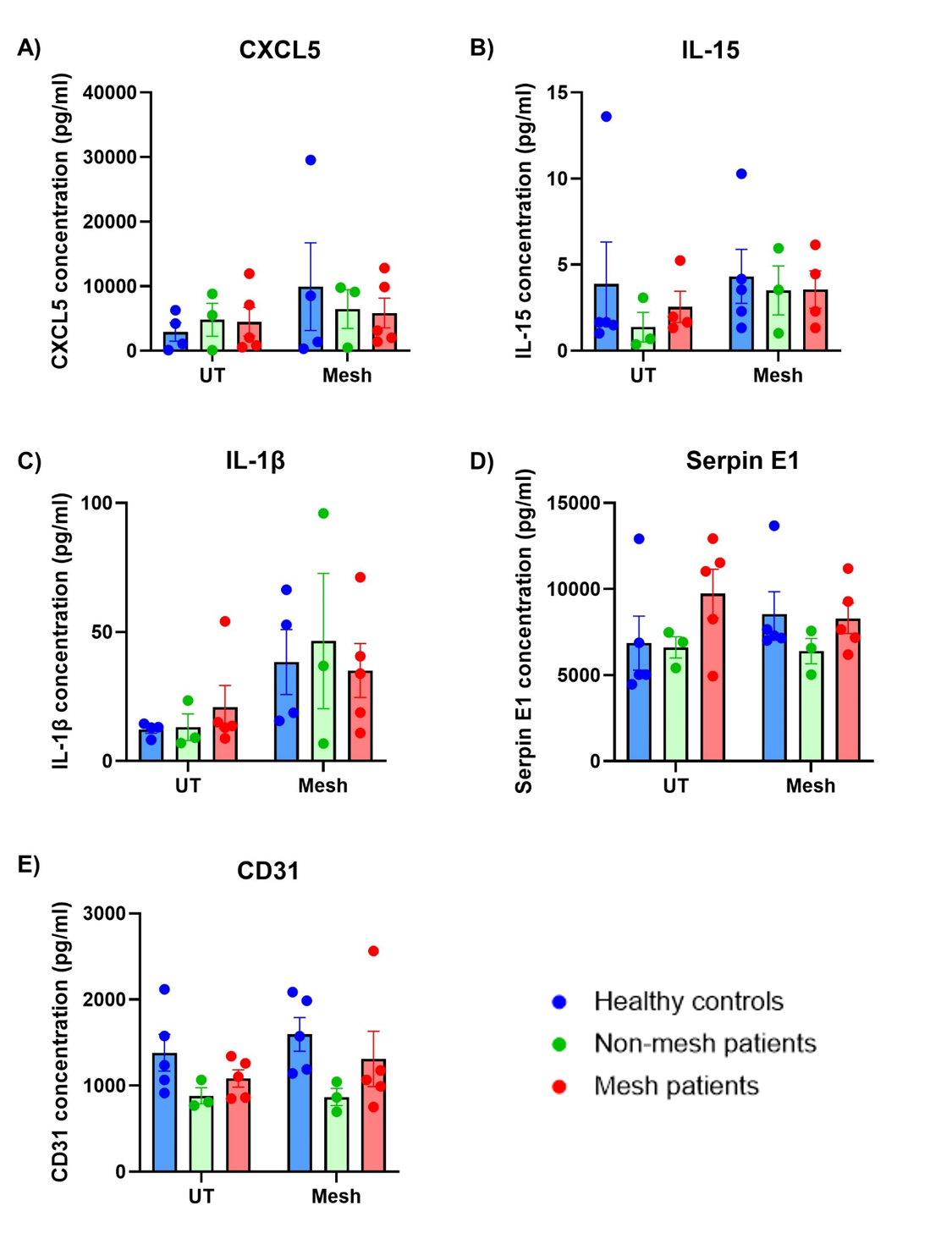
**

**Supplementary 1. Levels of immunomodulatory proteins secreted by PBMCs.** No notable differences were observed in the secretion of (A) CXCL5, (B) IL-15, (C) IL-1b, (D) Serpin E1, or (E) CD31 in both the untreated groups or upon re-exposure to mesh. Statistical analysis performed using a two-way ANOVA with Dunnett’s multiple comparisons, error bars represent the standard error of the mean (SEM).


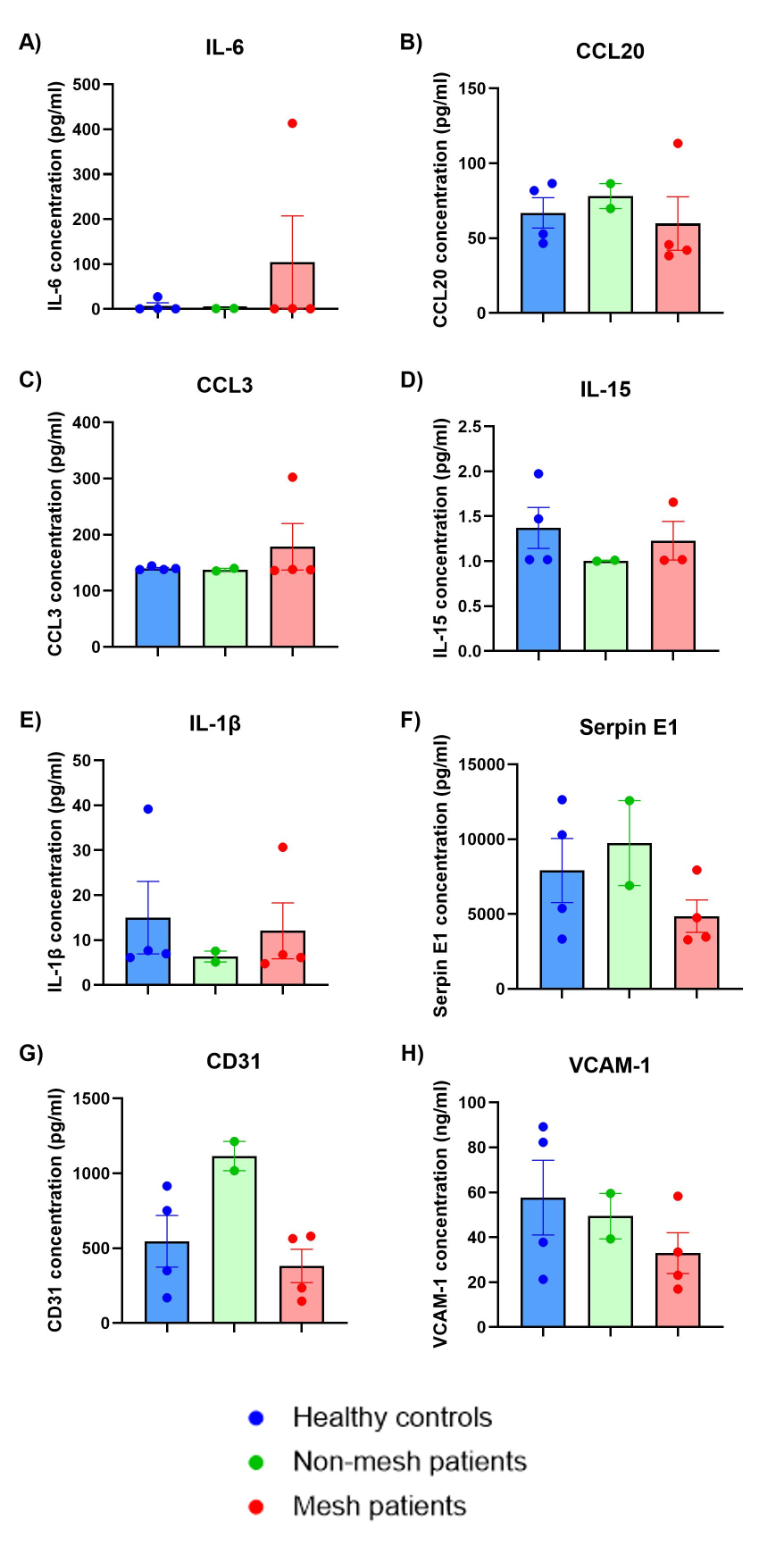


**Supplementary 2. Levels of immunomodulatory proteins in serum.** No notable differences were observed in the serum levels of (A) IL-6, (B) CCL20, (C) CCL3, (D) IL-15, (E) IL-1b, (F) Serpin E1, (G) CD31, or (H) VCAM-1 when comparing between healthy control, mesh, and non-mesh cohorts. Statistical analysis performed using a one-way ANOVA with Dunnett’s multiple comparisons, error bars represent the standard error of the mean (SEM).
